# Trends in total fertility rate in Ghana by different inequality dimensions from 1993 to 2014

**DOI:** 10.1186/s12905-022-01629-w

**Published:** 2022-02-23

**Authors:** Ebenezer Agbaglo, Pascal Agbadi, Justice Kanor Tetteh, Edward Kwabena Ameyaw, Collins Adu, Jerry John Nutor

**Affiliations:** 1 Department of English, University of Cape Coast, Cape Coast, Ghana; 2 Department of Nursing, College of Health Sciences, Kwame Nkrumah University of Science and Technology, Kumasi, Ghana; 3 Department of Population and Health, University of Cape Coast, Cape Coast, Ghana; 4 School of Public Health, Faculty of Health, University of Technology Sydney, Sydney, Australia; 5 Department of Health Promotion, Education and Disability Studies, Kwame Nkrumah University of Science and Technology, Kumasi, Ghana; 6 Department of Family Health Care Nursing, School of Nursing, University of California, San Francisco, 2 Koret Way, Suite N431G, San Francisco, CA 94143 USA

**Keywords:** Demographic and Health Surveys, Ghana, Global health, Inequality, Total fertility, Low-middle income countries

## Abstract

**Background:**

The past few decades witnessed a considerable decline in total fertility rates globally. However in Ghana, there has been a slight increase in the fertility rate with little understanding of the reason for the increment. To understand this change, it is important to first examine the trend over a considerable period of time while taking into consideration some important inequality dimensions. This informed the need for this present study as we examined the trends in total fertility rate in Ghana by different inequality dimensions from 1993 to 2014.

**Methods:**

Data from the 1993–2014 Ghana Demographic and Health Surveys were used for the study, and we relied on the World Health Organization’s (WHO) Health Equity Assessment Toolkit (HEAT) software for the analysis. The analysis involved disaggregation of TFR by wealth index, education, place of residence and region. This was followed by the estimation of inequality by Difference, Population Attributable Risk, Ratio and Population Attributable Fraction. In the analysis, we set the statistical significance at a 95% confidence interval.

**Results:**

For all surveys, the total fertility rate was consistently highest among the poorest women (7.00, 6.28, 6.77, 6.61 and 6.29 in 1993, 1998, 2003, 2008 and 2014, respectively). The highest total fertility rate was recorded among women with no formal education in all the survey years. For instance, in the 2014 survey, the total fertility rate for women with no formal education was 5.98 and those with secondary/higher had a total fertility rate of 3.40. Women in rural areas had a higher total fertility rate compared to those in urban areas (4.90 vs. 3.40 in 2014). In terms of sub-national regions, the Northern region was the region where women consistently had the highest total fertility rate.

**Conclusion:**

There is a need for a collective effort to design interventions and policies to create awareness among the people of Ghana especially girls and women on the implications of high fertility.

## Introduction

The past few decades witnessed a considerable decline in total fertility rates over the world. In 2019, the global fertility rate stood at 2.5 births per woman, indicating a decline from 2.8 births per woman from the year 2000 [1]. In sub-Saharan Africa, this decline has been much slower [2]. For instance, whereas the total fertility rate (TFR) in SSA only declined from 6.57 births per woman in 1950 to 4.62 births per woman in 2019, TFR of Asia and Latin America declined sharply from 5.83 and 5.83 births per woman in 1950 to 2.15 and 2.04 births per woman in 2019 respectively [3, 4]. This is critical, given the alarming impacts of uncontrolled rapid population growth on the wellbeing of nations [5]. Although the high population may have some virtue, for instance; increasing workforce, its negative consequences appear to preponderate the positive ones [6]. For instance, with rapid population growth, there is pressure on social amenities as well as unemployment, which has negative implications for the growth and development of low- and middle-income countries such as Ghana. In South Africa for example, it is reported that rapid population growth and large increases in school-age population has undermined efforts to improve quality of education and in Mozambique, it is estimated that only 30% of the population has access to health services due to rapid population growth [7].

To offset the negative implications of high rapid population growth, low- and middle-income countries worldwide are putting in measures in an attempt to check uncontrolled rapid population growth. This involves the institution of policies and interventions. In 2015, the United Nations adopted the 2030 Agenda for Sustainable Development emphasizing universal access to a full range of reliable and safe family planning methods to help people to decide responsibly and freely the number and spacing of their children [1]. The international community has therefore sought to expand contraceptive utilization, counseling, information dissemination and other family planning services as one of the tools to check uncontrolled rapid population growth [2, 8, 9]. The increased patronage of such services and programs have resulted in improvements in health-related outcomes such as reductions in maternal and infant mortality, unintended and high-risk pregnancies, and improvements in economic and schooling outcomes, especially for girls and women.

Although the fertility rates of most high-income countries are declining, the programs and interventions seem not to be working in many SSA countries as the rate of fertility is either stable, reducing at a slower pace, or is on the rise [8, 10–12]. For example, In Ghana it is reported that the TFR has been fluctuating over 2 decades [13, 14]. Despite the introduction of many government policies, programs and interventions such as the 2004 National Population Policy, Ghana Population and AIDS project (1996–2000), 2002 Free Compulsory Universal Basic Education (FCUBE) Contraceptive Social Marketing project (1987–1990), Ghana Family Planning and Health Programme (1990–1996) and Free Senior High School programs, TFR has not seen major declines and it remains unclear the trend over the past few years.

Research has revealed some association between fertility and other variables such as educational background [15], unmet need for family planning [15] and contraceptive use [16] and the 2019 World Fertility Policy document has also illustrated that variations in the trends in fertility across countries are associated with the trend in growing national wealth, educational expenditures, and income inequalities [1]. Perhaps, these variables could also play critical roles in Ghana’s TFR trend.

According to the Ghana Demographic and Health Survey (GDHS) [14], there have been fluctuations in the fertility rate in the country, and the World Bank has also reported that Ghana’s TFR is declining at a much slower pace [14]. The rate of decline since 1980 has been reducing sharply. For example, between 1980 and 1990 the rate of decline was 0.937, between 1990 and 2000 the rate of decline was 0.776, between 2000 and 2010 the rate of decline was 0.553 and finally, between 2010 and 2019 the rate of decline was 0.457 [17]. This implies that even though TFR in Ghana has been declining over the years, the rate of decrease has also been decreasing sharply. These are at the backdrop of several interventions Ghana has implemented over the past 3 decades to curb the rapid population growth such as the FCUBE in 2002, Free SHS in 2017, introduction of Community Health nursing to scale up provision of family planning services in 1960, introduction of subsidies on the importation of contraceptives in 2002 and the adoption of the 2004 National Population Policy.

To better understand the TFR situation in Ghana and what needs to be done to ensure stable and sustainable decline, there is the need to first examine the TFR trend over a longer period while taking into consideration some inequality dimensions. Hence, this study was conducted to examine the trends in total fertility rate by different inequality dimensions between 1993 and 2014. Findings from such a nationally representative study will help formulate and strengthen programs, strategies, and interventions to check uncontrolled rapid population growth in Ghana.

## Materials and methods

### Study design

The study relied on data from 1993, 1998, 2003, 2008 and 2014 versions of the GDHS. Generally, Demographic and Health Surveys are conducted in five years intervals in low-and middle-income countries under the MeasureDemographic and Health Survey (DHS) program. A stratified sampling technique involving two stages was used in collecting data in the various rounds of the GDHS. The first stage involved the division of the country into enumeration areas while in the second stage, households are selected from the enumeration areas. For a more detailed explanation of the methods, consult the final reports of the Ghana DHS [14]. In this study, women aged 15–49 were considered as the unit of analysis.

### Measures of inequality

The present study estimated women’s total fertility rate (TFR), measured as the total number of children a woman gives birth to. The analysis focused on economic status, education, place of residence, and subnational region. Economic status was estimated by the variable, wealth index, which is calculated by the principal component analysis (PCA) technique [18] in the DHS. The principal component analysis technique was used to compute wealth index taking into consideration the housing and household characteristics such as car, fridge and building materials. Wealth index consisted of poorest, poorer, middle, richer, and richest. No education, primary, and secondary/higher constituted women’s level of education. Place of residence was defined as urban vs. rural and subnational region referred to the then ten regions of Ghana. Rural/urban status was according to the definition by Ghana Statistical Service. According to Ghana Statistical Service, a place is considered as rural if the population is below 5,000 and urban if it is above 5,000.

### Data analysis

The estimation of inequality in TFR involved two steps. In the first step, we disaggregated TFR by economic status, level of education, place of residence, and subnational region. We then assessed inequality by Difference, Population Attributable Risk (PAR), Population Attributable Fraction (PAF), and Ratio, following an established practice in the scientific literature [19, 20]. The Difference and Ratio are simple measures whilst PAR and PAF are complex measures. The analysis was conducted using the WHO’s HEAT software version 3.1 [21]. Difference (D) was calculated as the disparity in TFR of “un-educated” group and “secondary/higher education” for education, the poorest group and the richest group for economic status, rural and urban populations for place of residence, as well as highest estimate and the lowest estimate for region. A more detailed explanation of the analysis procedures is provided elsewhere [19, 22].

PAR was computed by estimating changes in TFR for the reference sub-group, *yref,* and the national average of TFR. For ordered dimensions, y_ref_ is described by the most-disadvantaged sub-group, which is represented by those without formal education, and the poorest sub-groups. In the case of such binary dimensions as sex, y_ref_ indicates the sub-group with the lowest estimate, and in the present study, that subgroup is female. With regard to non-ordered dimensions such as sub-national region, y_ref_ denotes the subgroup with the lowest estimate. In calculating the PAF, we divided the population attributable risk (PAR) by the national average μ and multiplied the fraction by 100 (PAF = [PAR/μ] * 100). Higher levels of inequality are determined using greater absolute PAR and PAF values, whereas zero indicates the absence of inequality. The change in TFR over time was assessed concerning the 95% Uncertainty Intervals (UI) of the different survey years. Whereas an absence of overlapped UIs portrays the statistically significant difference between the two UIs, an overlap of UIs is evidence of inequality.

### Ethics approval and consent to participate

The study followed the Declaration of Helsinki for research involving human subjects. This was secondary data analysis; therefore, ethical clearance was not required from the authors of this study. However, all the GDHS surveys report that the surveys were approved by ICF International and the Ghana Health Service Research Ethics Committee. The Measure DHS Program also made sure the survey protocols complied with the U.S. Department of Health and Human Services regulations for the protection of human subjects. Especially, both written and informed consent was obtained before data were collected from the women. Minors were not included in this study.

## Results

### Trends in total fertility rate by different inequality dimensions, 1993–2014

Overall, TFR in Ghana decreased significantly from 5.50 to 4.14 between 1993 and 2008 but increased slightly to 4.15 in 2014. Throughout the surveys, there was a disparity in TFR across the four inequality dimensions. Specifically, TFR was consistently highest among the poorest women (7.00, 6.28, 6.77, 6.61 and 6.29 in 1993, 1998, 2003, 2008 and 2014, respectively), compared to the richest women (3.50, 2.45, 2.72, 2.45 and 2.85 in 1993, 1998, 2003, 2008 and 2014, respectively). Similarly, the highest TFR was recorded among women with no formal education in all the surveys, compared to those with secondary or higher education. For instance, in the 2014 survey, whereas women with no formal education had a TFR of 5.98, those with secondary or higher had a TFR of 3.40. The rural–urban disparity in TFR was also observed, with women in rural areas having a higher TFR, compared to those in urban areas (4.90 vs. 3.40 in 2014). In terms of regions, the Northern region was the region where women consistently had the highest TFR over the surveys (Table 1).

**Table 1:** Trends in total fertility rate, disaggregated across four inequality dimensions, 1993–2014

Dimension	1993 (5.50) N = 20,838		1998 (4.55) n = 21,817		2003 (4.58) n = 25,421		2008 (4.14) n = 21,922		2014 (4.15) n = 42,569	
	n	R [UI]	n	R [UI]	N	R [UI]	n	R [UI]	n	R [UI]
**Economic status**										
Poorest	3333	7.00 [5.95–8.22]	4506	6.28 [5.54–7.12]	4410	6.77 [5.67–8.09]	3501	6.61 [5.51–7.94]	6593	6.29 [4.86–8.12]
Poorer	4047	6.60 [5.62–7.74]	3748	5.49 [4.76–6.34]	4290	6.07 [5.18–7.12]	3967	5.12 [4.51–5.80	7194	5.44 [4.50–6.58]
Middle	4162	6.04 [5.25–6.94]	3971	5.05 [4.38–5.83]	4736	4.91 [4.16–5.80]	4347	3.94 [3.32–4.66]	8830	3.90 [3.28–4.63
Richer	4395	4.91 [4.26–5.66]	4348	3.38 [3.39–4.47]	5537	3.48 [2.98–4.05	5002	3.48 [2.99–3.99]	9766	3.31 [2.70–4.06]
Richest	4899	3.50 [3.09–3.97]	5243	2.45 [2.14–2.81]	6445	2.72 [2.33–3.19]	5102	2.45 [2.06–2.92]	10,185	2.85 [2.24–3.64]
**Education**										
No education	7661	6.67 [5.74–7.75]	6742	5.83 [5.17–6.58]	7686	6.02 [5.17–7.01]	5030	6.10 [5.25–7.08]	8775	5.98 [4.64–7.71]
Primary	11,030	5.14 [4.71–5.62]	3920	4.94 [4.39–5.56]	4855	5.47 [4.79–6.25]	4316	4.91 [4.29–5.63]	7202	4.90 [4.28–5.62]
Secondary +	2147	2.90 [2.42–3.47]	11,154	3.56 [3.23–3.93]	12,879	3.30 [2.95–3.69]	12,575	3.06 [2.77–3.38]	26,592	3.40 [2.90–3.99]
**Place of residence**										
Rural	13,137	6.36 [6.09–6.65]	14,010	5.41 [5.17–5.67]	13,274	5.83 [5.56–6.12]	11,241	5.03 [4.71–5.37]	19,425	5.10 [4.85–5.37]
Urban	7701	3.99 [3.68–4.32]	7807	2.96 [2.71–3.23]	12,147	3.18 [2.94–3.44]	10,681	3.21 [2.99–3.44]	23,144	3.36 [3.15–3.57]
**Region**										
Western	1792	5.54 4.86–6.30]	2616	4.70 [4.07–5.42]	2445	4.75 [4.01–5.63]	2001	4.06 [3.47–4.74]	4660	3.69 [3.23–4.23]
Central	2006	5.57 [5.07–6.12]	2476	4.78 [4.24–5.38]	1888	5.03 [4.21–6.01]	1846	4.94 [4.38–5.57]	4285	4.56 [3.99–5.21]
Greater Accra	2799	3.56 [3.10–4.08]	3613	2.66 [2.32–3.06]	4219	3.03 [2.60–3.47]	3872	2.70 [2.39–3.04]	8845	2.86 [2.62–3.13]
Volta	2230	5.41 [4.80–6.10]	2400	4.44 [3.89–5.07]	2195	4.30 [3.67–5.04]	1917	3.99 [3.46–4.61]	3288	4.15 [3.65–4.72]
Eastern	2337	5.10 [4.56–5.72]	2862	4.41 [3.91–4.97]	2714	4.32 [3.71–5.03]	2122	3.71 [3.26–4.23]	4017	4.17 [3.73–4.66]
Ashanti	3467	5.60 [5.12–6.12]	3339	4.76 [4.17–5.42]	5055	4.33 [3.83–4.90]	4514	3.80 [3.33–4.33]	8083	4.15 [3.79–4.55]
Brong Ahafo	2119	5.46 [4.57–6.53]	1549	5.40 [4.72–6.17]	2518	5.06 [4.45–5.74]	1921	4.44 [3.963–4.99]	3389	4.60 [4.17–5.06]
Northern	2046	7.39 [6.74–8.12]	1096	6.98 [6.07–8.01	2309	7.04 [6.28–7.89]	2087	6.96 [6.17–7.86]	3503	6.50 [600–7.05]
Upper West	729	6.02 [5.15–7.03]	549	6.14 [5.43–6.93]	694	5.57 [4.75–6.54]	1106	4.28 [3.73–4.92]	1551	4.83 [4.42–5.29]
Upper East	1309	6.44 [5.59–7.43]	1313	4.98 [4.35–5.69]	1380	5.08 [4.28–6.01]	532	5.02 [4.40–5.72]	945	5.24 [4.58–5.98]

R: Rate; UI: Uncertainty Interval

### The magnitude of TFR based on the summary measures

We found an extensive absolute and relative wealth-related inequality in TFR from 1993 to 2014 both by simple (D, R) and complex (PAF, PAR) measures. A case in point is that in the 2014 survey, the PAF (PAF = − 31.13, 95% CI − 38.27, − 24.00) and Difference measure (D = 3.43, 95% CI; 1.68, 5.18) indicated a significant wealth-related inequality. In that same year, there was a significant education-related inequality, which was both absolute (PAR = − 0.78, 95% CI − 0.94, − 0.63) and relative (R = 1.76, 95% CI; 1.23, 2.28). These findings indicate that both wealth and education favour women who are well-off in terms of economic and educational attainment, with respect to fertility. Similarly, we found absolute and relative urban–rural inequality in TFR from 1993 to 2014 both by simple (D, R) and complex (PAR, PAF) measures with a decreasing pattern. In the 2014 survey, the Ratio measure (R = 1.52, 95% CI 1.40, 1.64) indicated huge relative pro-urban disparities in TFR with over time decreasing pattern. We also found absolute (D, PAR) and relative (R, PAF) inequality in TFR across sub-national regions over the period studied. For instance, in the most recent survey, the PAR measure (D = 3.64, 95% CI 3.05, 4.22) and the PAF measure (PAF = − 31.05, 95% CI − 38.81, − 23.30) indicated substantial absolute and relative regional inequality between the region with the highest TFR (Northern region) and the one with the lowest TFR (Greater Accra region) (see Table 2).

**Table 2:** Inequality indices estimates of the factors associated with the total fertility rate (births per woman), 1993–2014

Dimension	1993			1998			2003			2008			2014		
	Est	LB	UB	Est	LB	UB	Est	LB	UB	Est	LB	UB	Est	LB	UB
**Economic status**															
D	3.49	2.28	4.71	3.83	2.98	4.69	4.05	2.77	5.32	4.16	2.89	5.44	3.43	1.68	5.18
PAF	− 35.93	− 44.62	− 27.24	− 45.87	− 54.52	− 37.22	− 40.29	− 48.33	− 32.26	− 40.55	− 50.13	− 30.97	− 31.13	− 38.27	− 24.00
PAR	− 1.96	− 2.44	− 1.49	− 2.08	− 2.47	− 1.68	− 1.84	− 2.21	− 1.47	− 1.67	− 2.07	− 1.28	− 1.29	− 1.59	− 0.99
R	2.00	1.59	2.41	2.56	2.09	3.04	2.49	1.90	3.07	2.70	2.02	3.38	2.20	1.43	2.98
**Education**															
D	3.77	2.65	4.90	2.27	1.49	3.06	2.72	1.74	3.71	3.04	2.08	3.99	2.57	0.96	4.19
PAF	− 47.02	− 59.74	− 34.31	− 21.09	− 26.96	− 15.22	− 27.29	− 32.68	− 21.91	− 25.75	− 31.24	− 20.21	− 18.72	− 22.35	− 15.08
PAR	− 2.57	− 3.27	− 1.88	− 0.95	− 1.22	− 0.69	− 1.24	− 1.48	− 0.99	− 1.06	− 1.29	− 0.83	− 0.78	− 0.94	− 0.63
R	2.30	1.76	2.84	1.64	1.38	1.89	1.83	1.48	2.17	1.99	1.63	2.35	1.76	1.23	2.28
**Place of residence**															
D	2.37	1.95	2.80	2.45	2.09	2.80	2.65	2.28	3.03	1.82	1.43	2.22	1.74	1.41	2.08
PAF	− 27.26	− 34.07	− 20.46	− 34.74	− 41.99	− 27.48	− 30.35	− 35.92	− 24.75	− 22.56	− 28.91	− 16.20	− 19.17	− 23.34	− 15.00
PAR	− 1.50	− 1.87	− 1.12	− 1.58	− 1.90	− 1.25	− 1.38	− 1.64	− 1.13	− 0.93	− 1.20	− 0.67	− 0.80	− 0.97	− 0.62
R	1.59	1.45	1.74	1.83	1.65	2.01	1.83	1.66	2.00	1.57	1.42	1.72	1.52	1.40	1.64
**Region**															
D	3.84	3.00	4.68	4.31	3.28	5.35	4.03	3.12	4.94	4.27	3.36	5.17	3.64	3.05	4.22
PAF	− 34.99	− 47.03	− 22.95	− 41.25	− 52.31	− 30.20	− 34.54	− 45.17	− 23.91	− 34.96	− 46.59	− 23.33	− 31.05	− 38.81	− 23.30
PAR	− 1.91	− 2.57	− 1.26	− 1.87	− 2.37	− 1.37	1.59	− 2.07	− 1.10	− 1.45	− 1.93	− 0.97	− 1.29	1.61	− 0.97
R	2.08	1.74	2.42	2.62	2.11	3.13	2.34	1.91	2.77	2.58	2.14	3.02	2.27	2.00	2.54

Est: Estimate; LB: Lower bound; UB: Upper bound

## Discussion

This study aimed at examining critically the trends in TFR by different inequality dimensions between 1993 and 2014. Overall, TFR in Ghana decreased significantly from 5.50 to 4.14 between 1993 and 2008 but increased to 4.15 in 2014. Corresponding with the findings of the present study, Asamoah et al. [22] revealed a decrease in total fertility rates from 1988 to 2008. The present study also revealed disparities in total fertility rate for all the four indicators across the period under focus. Thus, variations exist among women with respect to wealth status, education, place of residence and region. The findings imply that distinct fertility interventions may be required for different category of women based on their wealth and educational status as well as place of residence and region. This finding accords with Finlay et al.’s [23] findings that there existed inequalities in total fertility rates in Ghana between 1990 and 2014. Similarly, a study by Asamoah et al. [22] also revealed an increase in education and income-related inequalities in TFR. Having discussed the trends in TFR across the period, we now discuss the findings concerning the four inequality dimensions used in the present study: wealth, education, place of residence, and region.

The study revealed consistent wealth-related inequality in favour of the richest women across the survey periods. Specifically, the TFR was consistently highest among the poorest women (7.00, 6.28, 6.77, 6.61 and 6.29 in 1993, 1998, 2003, 2008 and 2014, respectively), compared to the richest women (3.50, 2.45, 2.72, 2.45 and 2.85 in 1993, 1998, 2003, 2008 and 2014, respectively). This finding accords with previous research in Ghana [22]. In a related previous study in Ghana, Asamoah et al. [23] also observed high rates of fertility among the poorest women. They explained that the richest women could utilize pricy, long-term contraceptives to delay childbirth. Askew et al. [24] similarly noted that while wealthy women may have the means to reduce their fertility rates, poorer women may not, due to financial constraints.

Similar to the findings of some previous studies [22, 25, 26], the highest TFR was recorded among uneducated women in all the survey years, compared to those with secondary/higher education. A previous study by Asamoah et al. [22] revealed a significant reduction in fertility rate among educated women over the years. Another study by Abdul-Salam et al. [27] in Ghana revealed that women’s estimated number of children is dependent on their level of education. This finding is not surprising given the socio-economic empowerment education gives to women. Thus, educated women are also more likely to know the benefits of taking care of their health and that of their children. It is also established that such women often delay their marriages, prefer fewer children, and use family planning methods such as contraception [25, 26]. This finding, therefore, highlights the need to focus on education to reduce total fertility rates in Ghana.

Finally, the present study observed rural–urban disparity in TFR with women in rural areas having a higher TFR compared to those in urban areas (4.90 vs. 3.40 in 2014). Possibly TFR among the poor is high because their perceive children as a source of security and livelihood in their old age or due to the cost of contraception. This finding corroborates the findings of some other studies [22, 24, 25]. In explaining this finding, Agyei-Mensah and Owoo [28] intimated that the high cost of living in urban areas may force residents into opting for smaller families. Urban residents may also be more educated and characterized among the richest quintile and this could affect their decision making on family sizes. Relatedly, the study reveals cross-regional variations in total fertility rates in Ghana, with the Northern region which is predominantly rural consistently having the highest TFR throughout the survey. According to Agyei-Mensah and Owoo [28], this variation may be explained within the context of different cultural and religious factors that characterize each region in Ghana. The authors noted that certain tribes in northern Ghana still place a premium on large family size, basically as a result of historical antecedents, such as wars which required huge populations of energetic youth.

### Strength and limitation of the study

This study followed a repeated cross-sectional study design and as such causal inference cannot be made. Despite this limitation, the study provides a nationally representative coverage of women’s total fertility rate with the combination of a simple, complex, relative, and absolute measures to provide a comprehensive analysis of the inequality of in TFR of Ghana..

## Conclusion

This study examined critically the trends in total fertility rate by different inequality dimensions between 1993 and 2014 in Ghana. Specifically, the study has revealed that women living in rural areas, those with no formal education, and those among the poorest wealth index have a higher total fertility rate. Thus, those women are likely to desire for more children. This calls for a collective effort by major stakeholders in Ghana to accelerate girl child education programs as this will ensure that future women in Ghana will be formally educated, which will open them up to good job opportunities that will also empower them financially to make informed family size decisions. Again, this is also important because it is believed that as girls stay in school much longer, their reproductive age/period is reduced. Besides, there is the need to involve both men and women in TFR interventions.

## Data Availability

The Ghana DHS data supporting the analysis of this study is available in the DHS repository. The DHS datasets are available for free after a simple registration process at https://dhsprogram.com/data/dataset_admin/index.cfm.

## Abbreviations

AIDS: Acquired Immune Deficiency Syndrome
CI: Confidence interval
DHS: Demographic and Health Survey
EA: Enumeration areas
GDHS: Ghana Demographic and Health Survey
HEAT: Health Equity Assessment Toolkit
PAF: Population attributable fraction
PAR: Population attributable risk
PCA: Principal component analysis
SSA: Sub-Saharan Africa (SSA)
TFR: Total fertility rate
UI: Uncertainty interval
WHO: World Health Organization
